# The changing view of insulin granule mobility: From conveyor belt to signaling hub

**DOI:** 10.3389/fendo.2022.983152

**Published:** 2022-09-02

**Authors:** Bastian Gaus, Dennis Brüning, Sofie Groß, Michael Müller, Ingo Rustenbeck

**Affiliations:** ^1^ Institute of Pharmacology, Toxicology and Clinical Pharmacy, Technische Universität Braunschweig, Braunschweig, Germany; ^2^ Institute of Dynamics and Vibrations, Technische Universität Braunschweig, Braunschweig, Germany

**Keywords:** actin, calcium, insulin granules, insulin secretion, pancreatic islet, TIRF microscopy

## Abstract

Before the advent of TIRF microscopy the fate of the insulin granule prior to secretion was deduced from biochemical investigations, electron microscopy and electrophysiological measurements. Since Calcium-triggered granule fusion is indisputably necessary to release insulin into the extracellular space, much effort was directed to the measure this event at the single granule level. This has also been the major application of the TIRF microscopy of the pancreatic beta cell when it became available about 20 years ago. To better understand the metabolic modulation of secretion, we were interested to characterize the entirety of the insulin granules which are localized in the vicinity of the plasma membrane to identify the characteristics which predispose to fusion. In this review we concentrate on how the description of granule mobility in the submembrane space has evolved as a result of progress in methodology. The granules are in a state of constant turnover with widely different periods of residence in this space. While granule fusion is associated +with prolonged residence and decreased lateral mobility, these characteristics may not only result from binding to the plasma membrane but also from binding to the cortical actin web, which is present in the immediate submembrane space. While granule age as such affects granule mobility and fusion probability, the preceding functional states of the beta cell leave their mark on these parameters, too. In summary, the submembrane granules form a highly dynamic heterogeneous population and contribute to the metabolic memory of the beta cells.

## Introduction: Techniques shape hypotheses - how the present views on insulin granule mobility emerged

The views on the fate of the insulin granule were and are largely determined by the measuring techniques available. For decades the dominant technique was the electron microscopy and the observations made by it have led to the terminology still in use today. A “docked” granules is a granule the membrane of which is so close to the plasma membrane that no empty space is visible in between (1). This close vicinity was and still is widely regarded as a precondition for the fusion reaction. The gain of functional competence to undergo the fusion reaction was separated from the topological aspect by naming it “priming” (2).

The conventional view is that priming occurs after docking and that a “docked and primed” granule awaits the fusion signal in the form of a rising Ca2+ concentration in its immediate vicinity (3). Although electron microscopy can in principle document fusions at the single granule level, it does not permit time resolution since only end point measurements are possible. Also, the documentation of fusion reactions was hampered by the duration of the fixation step and remained rare until the use of the freeze-etching method (4–6).

The EM data were used to complement the kinetics of insulin secretion obtained from experiment with the perfused pancreas or perifused isolated islets. Of note, many concepts, which current investigations seek to prove or disprove have emerged from the interpretation of secretion data. This is particularly true for the concept that different compartments underlie the phasic nature of insulin secretion (7). While the concept of different pools of vesicles contributing to the release pattern of neurons or neuroendocrine cells implies their different localization (8, 9), the hypothesis of compartmentalization of insulin secretion does not necessarily describe the spatial organization of the granules, but was equally valid for purely functional differences or different metabolic activity acting on the granules. (10).

Among the early observations there is one which continues to generate considerable interest for the current imaging methodology: it is the preferential release of newly synthesized insulin and in consequence newly synthesized granules. It has been shown by radioactive labelling prior to stimulation that the ratio of labelled per total insulin was higher in the released insulin than in the remaining islet content of insulin (11–13). Furthermore, it was shown that the conditions during which the radioactive labelling took place, was of relevance for the preferential release (14). But the question how the prefusion behavior of the young granules differs from the one of the pre-existent granules could only be addressed by the new techniques of quantitative live-cell imaging.

A more dynamic view on the exocytosis of insulin granules emerged with the high resolution measurements of membrane capacitance enabled by patch-clamping with a lock-in amplifier (15). The increase in cellular membrane capacitance is caused by the addition of the granule membrane surface to the total surface of the beta cell plasma membrane when the fusion pore is generated. This permits measurements of granule fusion at a single granule level. However, this technique reports only the opening of the fusion pore, what happens before and what happens afterwards has to be indirectly deduced from the variation of the experimental conditions and leaves room for interpretation. This in turn is influenced by the pre-existing range of hypotheses. E.g. the hypothesis that one fusion equals one complete release of content may be generally valid for neuronal exocytosis (16), but may be of limited validity for the release of hormones.

While a clear advantage of the capacitance measurements is that it is label-free, it is relevant to note that the fusion reaction is not only generated by physiological or pharmacological stimuli, but is elicited by a train of depolarizations imposed on the cell. The choice of amplitude and duration of the depolarization is of decisive influence on the evoked reactions (17). This may be the reason why extremely high rates, like 500 fusions per second were initially reported (18). Another drawback of this technique is that upon prolonged stimulation endocytosis sets in and counteracts the increase in membrane capacity. The rate of endocytosis was found to be much slower, about 9 granules per second, still high, but closer to the range of secretion as assessed by measuring the amount of released insulin. Thus, it was hypothesized that the rate of endocytosis might be a limiting factor for granule fusion upon prolonged stimulation (18). From a conceptual viewpoint these observations are interesting in that they suggest that the ability of the beta cell to perform fusion reactions may not be rate-limiting for the achievable secretion rate, at least not within the physiological range. The limiting function could rather be exerted at preceding steps which may gate the access to the fusion sites (see below, chapter on cortical actin).

Taken together, these examples illustrate the point that with each new technique it is worthwhile to reconsider the hypothetical framework inherited from the limited perspectives of the preceding techniques.

## Questions to be answered by live cell imaging of insulin granules

The fundamental question of research on stimulus-secretion coupling in the pancreatic beta cell is still the same as it was in the preceding decades: which mechanisms form the biphasic kinetics of glucose-induced (or, in a broader sense, nutrient-induced) insulin secretion?

The endocrine pancreas responds to an increase in ambient glucose with a biphasic pattern of insulin release, represented by a short first phase, followed by a transient nadir and a slowly increasing second phase release (19). While these features are generally known, it is less widely known that after prolonged periods of constant stimulation the secretory response decreases from a maximal to a moderately elevated level. During this “third phase” the stimulus-secretion coupling has become desensitized (20, 21).

The biphasic response pattern is best shown in response to a ‘square wave’ glucose stimulus, either *in vivo* or *in vitro* (22, 23). It is obvious that such a stimulation pattern is non-physiological, the glucose resorption in the gut yields a comparatively slowly ascending glucose concentration, which is much less effective to produce a first phase (24). Nevertheless, the biphasic response pattern is the hallmark of the healthy endocrine pancreas. A diminished insulin response, often described to be more prominent during the first phase, but also recognizable during the second phase is associated with the manifestation of type 2 diabetes or models of this disease (25–27). Consequently, many of the recent reports on insulin granule behavior include these pathophysiological aspects (for reviews see 28–30).

The importance of the questions to be answered by live cell imaging of granules differs according to the conceptual framework. Under the assumption that the functional subdivision into a readily releasable and a reserve pool corresponds to the subdivision into docked and more or less distal non-docked granules as in neuronal exocytosis (31, 32), the following questions appear relevant: Have all granules which fuse with the plasma membrane been in a docked state prior to fusion? Is the readily releasable pool identical with the pool or a subpool of the docked granules? Is the readily releasable pool the correlate of the first phase of insulin secretion? Does the refilling of the readily releasable pool involve the translocation of granules or is it purely functional? Is the requirement for docking uniform for all phases of insulin secretion?

If the compartmental hypothesis is considered in a wider context, such that it reflects the sequence of signals acting on the granules (10), a number of additional questions come up. How is the formation of granules related to the release? Is the transport route from the site of generation to the plasma membrane essentially the same for all granules or are the short cuts and detours? Once arrived at the plasma membrane, do the granules stay there until being released (or degraded) or is there a steady turnover? How are these processes modulated by beta cell nutrients? Why is there such a large surplus of insulin granules if only a small minority is released during a physiologically relevant time span? And, as a consequence of the latter, what distinguishes aged granules from newly formed granules?

Of course, answering these questions does not only require topological information, but also information on biochemical mechanisms. The task to integrate biochemical reactions into spatial and temporal coherence is currently made easier by the ever expanding range of genetically encoded fluorescent indicators.

## Live cell imaging to analyse granule behavior - methodological considerations

Different live cell imaging techniques have been employed to visualize insulin granules and to characterize their behavior. Arguably, the most influential technique thus far has been TIRF (total internal reflectance fluorescence) microscopy, followed by CLS (confocal laser scanning) microscopy, usually as the point scanning version, occasionally as the spinning disk version. Multiphoton microscopy has only rarely been used and super-resolution techniques like STED microscopy have yet to make their mark on insulin granule research.

All of the above require fluorescent labeling of the granules which may give additional information beyond the spatial dimension but which may also lead to artefactual observations and misinterpretations. While early investigations used small molecule labels of low specificity such as quinacrine (33), targeting fluorescent proteins to the granules by fusing them to granule-specific proteins has become standard practice. Problems of this approach are that the cargo-directed labels like EGFP linked to insulin (34, 35) or to C-peptide (36) may alter the intragranular processing and the release after fusion. Even small differences in the linker region between the label and the cargo protein were shown to influence the post-fusion characteristics of cargo release (37). Likewise, attaching the label to the transmembrane granule protein phogrin has been shown to disturb the attachment of the granule to and fusion with the plasma membrane (38, 39).

These considerations are also valid for the newer labelling techniques like SNAP-tag or Halotag (40), since the proteins to which the small fluorescent molecules irreversibly bind are of similar size (182 or 297 amino acids, respectively) as the EGFP (238 amino acids). The interpretation is particularly difficult when observations have to be compared which were obtained with different labels like EGFP and dsRedE5 (41). Finally, all fluorescent labels are prone to photobleaching, thus excitation energy and exposure time have to be carefully chosen to avoid misinterpretations (42).

The pH-dependence of the EGFP fluorescence (43), often considered as problematic, has been used as a surrogate marker for exocytosis, since the formation of the fusion pore increases the pH value of the acidic granule interior (44, 45), thereby de-quenching the EGFP fluorescence. The sudden increase of the fluorescent spot is easy to recognize, but is insufficient to conclude that the granule content has been released or in other words that complete exocytosis has taken place. Finally, it has to be taken into account that granule labeling by heterologous expression of fluorescent proteins may leave the older, pre-existing granules unmarked (46), thus creating a bias in the quantitative evaluation.

Light microscopy has a lower axial (z-dimension) than lateral (x/y dimension) resolution, this remains true even for the super-resolution techniques like STED microscopy. TIRF microscopy is the exception to this rule, however, this advantage comes at the price that only a layer of around 150 nm below the plasma membrane can be rendered visible. Depending on the angle of incidence of the laser beam, the calculated decay constant (reduction of the initial excitation intensity at the glass-membrane interface to 1/e = 37%) can be varied until the change into the epifluorescence mode occurs (47, 48). Two features of TIRFM have to be pointed out here. First, because of the exponential decay of excitation within the TIRF layer, small axial movements will result in marked changes of fluorescence. Second, a reduction to 37% is not necessarily equal to the complete loss of fluorescence detection, even when considering the inner filter effect of fluorescence emission. This is rather to be assumed at a reduction to 10% or about twice the decay constant.

To illustrate this point: the diameter of a granule is ca. 250 nm (49), so an axial movement towards the cell interior by about half a diameter is likely, by one diameter is certainly sufficient to let the granule fluorescence disappear. In consequence, examination of events beyond this distance, like granule transport from the trans-Golgi region to the plasma membrane, has to be performed by confocal or by multiphoton microscopy. Under ideal conditions, a lateral resolution of 150 nm can be achieved by confocal microscopy, but 200 nm is what can be considered as a typical value. The axial resolution, however, is only about 500 nm (50). The latter is about the double of a granule diameter, which seriously limits the reconstruction of 3D-trajectories.

Another factor to consider is that the time resolution of confocal microscopy is limited by the point scanning principle, which may require sampling rates of only 1 s^-1^ for well-resolved images (51). This limitation is much less severe with the spinning disk illumination, but until recently this technique suffered from low light intensity and pin-hole crosstalk (52). Simulations of granule movements, based on real TIRF data sets, showed that an acquisition frequency of 6 to 8 images per second was sufficient to for low error probabilities in tracking at the typical density of granules. A 99% correct assignment could be achieved at a nearest neighbor distance of 8 pixels, well below the average distance at this acquisition frequency, which was in the range of 10 -13 pixels (53). Currently, there is no technique that permits 3D measurements of the entire granule population of a primary beta cell at sufficient temporal and spatial resolution. However, this may change with the further development of light sheet microscopy.

## Granule mobility - a simple term for a complex issue

It is no question that TIRF microscopy is the technique of choice to study the molecular interactions between the insulin granule and its attachment site at the plasma membrane (e.g. 54, 55) or to study the release of granule content after fusion pore opening (e.g. 56, 57). If the aim of the study is to characterize granule mobility, the major advantage of TIRF, the shallow depth of field may also be a limiting factor. In the first investigations utilizing TIRF microscopy the fusing granules (as identified by EGFP fluorescence increase) were subdivided into those which were present from the beginning of the registration and those which appeared only during the course of the registration. The former were considered to be in the docked state, the latter were named newcomers (34). It was concluded that the fusions during the first phase of glucose stimulation and during KCl stimulation were mostly by docked granules, whereas the fusions during the second phase of glucose stimulation were predominantly by newcomers. In this study the role of the newcomers was postulated to consist in the refilling of the readily releasable pool by physical translocation (34). In contrast to KCl, sulfonylureas drugs were found to induce the fusion of newcomer granules (58), an observation for which a mechanistic rationale is still lacking.

In a later study which aimed at describing the mechanism underlying the enhancement of insulin secretion by cAMP-signaling the newcomer granules were subdivided (59). cAMP increased the fusion rates throughout the entire exposure to stimulatory glucose, thus an effect on both phases of secretion could be assumed. Intriguingly, the increase by cAMP was nearly completely due to newcomer granules which fused immediately after arrival in the TIRF layer. These were named restless newcomers, whereas granules which remained visible for a while before fusing (and were presumably docked during this phase) were name resting newcomers (59). The resting newcomers made up a small minority under all conditions, such as stimulation by glucose alone. Fusions by pre-docked granules (“old faces”, defined as being those which were present at the beginning of the registrations) prevailed during stimulation by very high extracellular potassium concentration (60 mM), but not during the initial phase of glucose stimulation with or without treatment to elevate cAMP (59, 60).

The view that the docking of the granules is an indispensable step prior to the fusion was further questioned by the observation that the knock-out of granuphilin, an effector of the small GTPase Rab27a, virtually abolished docking as shown by both, TIRFM and by EM, but did not diminish fusion and secretion, rather, both were enhanced (61). Consequently, it was hypothesized that docking is not a precondition for fusion, but rather a waiting state, perhaps even exerting a constraint on the probability of fusion (62). The subdivision of the granules into pre-existent granules, newly arrived granules which fuse after having stayed at the membrane for a while, and granules which fuse immediately after appearance corresponds largely to the one proposed by Seino´s group, even though a different terminology was used in these reports.

The heterodox view that fusion can occur without previous docking, at least in insulin secreting cells, was supported by two-photon imaging to simultaneously measure FRET signals of SNARE assembly and insulin exocytosis. These data suggest that SNARE proteins exist in multiple stable configurations, and Ca^2+^ influx triggers exocytosis by distinct mechanisms and distinct kinetics (63). Further investigations by these authors showed that, unlike in neurons, SNAREs are not yet assembled in resting beta cells and assemble only shortly before exocytosis, a fact reflecting the much lower rates of fusion in beta cells than in neurons (64).

The studies mentioned so far have in common that they focus on the fusion reaction and then reconstruct the preceding steps of those granule which have fused. Since the number of granules exceeds by far the number of granules which are released even during prolonged stimulation, it appeared relevant to characterize the number and behavior of the entirety of granules in the submembrane membrane space to look for characteristics which predispose for fusion or for continued residence or for return into the cell interior.

Such an encompassing description requires a larger number of parameters. In addition to the number of granules per time the cumulative number during the measuring interval as well as the rates of arrival and of departure (back into the cell interior) are needed to assess the turnover. Since the residence in the submembrane space is of variable duration, three categories were defined; short-term, medium term and long-term resident granules (53). Arrivals and departures essentially reflect the granule mobility in the z-dimension. To describe the mobility in the x/y-dimension in a time-resolved manner, the caging diameter was chosen (53, 65). In contrast to the much more widely used mean square displacement, the caging diameter does not require the assumption of a steady-state mobility.

Of all the granules which appeared in the submembrane space under steady state conditions, 82% were present for less than 1 s, 16% had a residence time between 1 s and 25 s and only 2% were present for a longer time. While the rate of arrivals and departures and, in consequence, the cumulative granule number and the number of short term residents increased in response to KCl- or glucose-stimulation, the mean caging diameter was not significantly affected (42). When only the fusing granules were evaluated, the caging diameter revealed differences between those granule which had newly arrived and those which were pre-existent. The first measurable caging diameter of newly arrived granules was significantly larger than the one of the pre-existent granules. The last caging diameter immediately before fusion showed that the lateral mobility of the newly arrived granules had decreased and become more uniform, but it was still significantly larger than the corresponding one of the pre-existent granules (42). This supports the above view that different combinations of fusion-regulating factors are possible during the pre-exocytotic state (64). Under the given conditions however, fusions by newly arrived granules made up only a minority (< 15%) of the total number of fusions (42).

**Figure 1:**
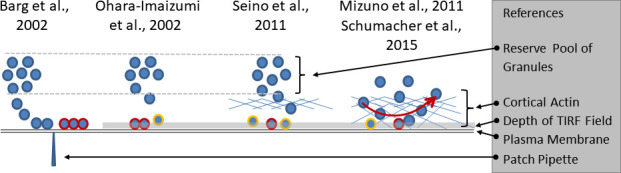
Growing complexity of the relation between granule mobility and fusion competence. The electrophysiological investigations (left part of the sketch) led to the definition of a readily releasable pool of docked and primed granules (highlighted by red rims), the emptying of which caused the nadir between the first and the second phase of insulin secretion. The slow refilling from a distant reserve pool was considered as the cause of the slower pace of secretion during the second phase. The initial TIRF studies (second from left) considered the predocked granules as the correlate of the readily releasable pool and noted an increasingly relevant contribution of newcomer granules (highlighted by yellow rims) with time. Later (second from right), a predominant contribution of restless newcomer granules to the first phase was described. The lower fusion rates during prolonged stimulation were ascribed to the passage through the cortical actin layer. The rightmost part of the sketch combines two sets of observations. The cortical actin layer, which extends to the immediate vicinity of the plasma membrane is not just an obstacle, but a near-membrane storage site of granules. There is a constant exchange of granules which arrive at the submembrane space and leave again after different periods of residence, mostly within one second. Fusion is possible after varying periods of residence.

Returns of granules back in to the cell interior have been noted early (34), but were apparently not considered relevant enough for a quantitative treatment. This was made possible by an observer-independent evaluation programme (53). Here, track endings that were not linked to any other track were termed “departures”, when the granule had left the evanescent field without fulfilling the criteria for exocytosis. The rate of departures closely mirrored the rate of arrivals both in MIN6 cells and in mouse beta cells (42, 66), even though the turnover rate was about 50% higher in MIN6 cells than in beta cells (**see** **Table 1**). So, granule mobility is not a unidirectional movement towards the plasma membrane, as often depicted in schematic drawings, but comprises a continuous exchange of the granules in the submembrane space (**Figure 1**).

**Table 1:** Typical values of the parameters to describe granule mobility in the submembrane space of MIN6 cells and primary mouse beta cells.

Parameter	MIN6 Cells labelled with hIns-EGFP	Beta Cells labelled with hIns-EGFP	Beta Cells labelled with mIns-C-emGFP
**Cell footprint area** **(square micrometers)**	332 ± 27	102 ± 13	168 ± 21
**Granule number per image** **(first image of the sequence)**	337 ± 34	175 ± 16	201 ± 26
**Cumulative granule number** **(per 25 s image sequence)**	6972 ± 873	2314 ± 207	2681 ± 269
**Mean turnover per sequence** **(Cumulative number/number per image)**	20.7	13.4	13.3
**Short-term resident granules** **(% of cumulative granule number)**	5720 (82%)	1874 ± 176 (79.8 ± 0.8%)	2153 ± 234 (80.3 ± 0.7%)
**Long-term resident granules** **(% of granule number first image)**	118 ± 12 (35 ± 4%)	75 ± 11 (43 ± 2%)	61 ± 14 (32 ± 3%)
**Arriving/Departing Granules (Difference between Images)**	8.8 ± 0,6%	6.3 ± 0.3%	7.0 ± 0.4%
**Caging Diameter** **(at Half-Maximal Abundance)**	124.5 ± 2.7 nm	82.5 ± 1.5 nm	85.5 ± 1.5 nm

Values are taken from 42 and from 66.

## Regulation of granule mobility by calcium

The decisive role of depolarization-induced Ca^2+^ influx for triggered exocytosis is beyond dispute. This is not only true for synaptic vesicles, but also for secretory granules, such as insulin granules in the beta cell (67). It is less clear as to whether the resulting increase in the cytosolic Ca2+ concentration is also a signal for granule transport to the cell periphery and for granule mobility in the submembrane space.

Early investigation have concluded that not Ca^2+^ influx, but rather Ca^2+^ release from internals stores is the mechanism by which glucose activates transport to the cell periphery (68, 69). While a role of Ca^2+^ release from the ER could be confirmed by CLSM, automated tracking identified a subgroup of fast moving granules which responded to Ca^2+^ influx by KCl (30 mM) depolarization (51). The authors concluded that the fast moving granules represent those which are destined to refill the readily releasable pool. Of note, KCl was more effective than glucose to elicit changes in granule mobility in both subgroups (51). Selective photoactivation of single granules, which permitted tracking by CLMS with a high spatial and temporal resolution, gave a somewhat different picture, in that glucose stimulation transformed granules of restricted mobility into fast moving granules with a directed movement. However, the mechanisms by which glucose brought about this transition were not further analyzed (70).

The most immediate mechanism to elicit a Ca^2+^ influx *via* VDCC is the KCl depolarization. As an experimental tool to stimulate the secretion of perifused islets KCl concentrations of 30 mM to 40 mM are used (71, 72), and 60 mM to 75 mM KCl are used to elicit exocytosis during live cell imaging. (62, 73). It is often stated that the secretory response to KCl depolarization is the equivalent of the first phase insulin secretion (see e.g. 72). Mechanistically, such an equivalence seems obvious for the depolarizing effect of pharmacological K_ATP_ channel closure (74), but the relation is more complicated for KCl depolarization.

The depolarization strength of 15 mM KCl is close to the one of KATP channel closure, but has only a modest transient effect on insulin secretion (74, 75). Higher KCl concentrations elicit progressively higher secretory responses without saturation and induce a desensitization to subsequent nutrient stimulation (75, 76). Correspondingly, the maximal fusion rates established by 60 mM KCl were higher than those produced by glucose stimulation (ca. 10 v. 1 per min and 200 μm^2^), but were less enduring (59, 60, 62). When measured by 2-photon microscopy in intact islets the fusion rate was 13 per cell and per minute during the first phase of glucose-induced insulin secretion (77). The above caveats notwithstanding, depolarization by high KCl concentration continues to be the preferred experimental technique to elicit insulin granule exocytosis.

The effect of Ca^2+^ influx on submembrane granule number and mobility in MIN6 cells was tested using depolarizations with 15 mM and 40 mM KCl. This led to the reduction of submembrane granules which were not so much due to the number of exocytoses, but to an increased turnover, as was visible by the increased numbers of arriving granules, short-term residents, and departures (35). Since the effect could be antagonized by nifedipine, Ca 2+ influx *via* L-type channels does not only trigger granule fusion but diminishes the granule number by accelerating the turnover.

In this context the observation is interesting that a difference apparently exists between the consequences of glucose stimulation and those of KCl stimulation: The total number of granules, the short term resident granules, and the arriving granules, which are all parameters of granule turnover, were significantly smaller for glucose than for 40 mM KCl (66). This may be part of the phenomenon that insulin secretion by KCl stimulation recedes the faster the higher the initially achieved secretion rates were (75, 76).

The slower and less extensive increase of the Ca^2+^ concentration in the submembrane space by 15 mM KCl as compared with 40 mM KCl was also used to test the hypothesis that granule pools of different Ca^2+^ sensitivity exist (78). In this hypothesis, the docked granules are of low Ca^2+^ sensitivity and require large increases of Ca^2+^ in their vicinity to fuse, whereas the newcomer granules, considered to be relevant for the second phase, are highly Ca^2+^ -sensitive and thus require a comparatively smaller increase (79, 80).

While the Ca^2+^ increase by 15 mM KCl is in fact lower than that of 40 mM KCl the resultant modest and transient increase in secretion bears little resemblance to a fully developed second phase. Also, the role of Ca^2+^ for the mobilization of the newcomer granules remained unclear. In a wider perspective, this hypothesis concurs with those by Kasai etal. (62) and Takahashi etal. (63) in that different combinations of preconditions can ultimately lead to exocytosis as opposed to a uniform sequence of events.

From a methodological stand point, the measurement of a conventional small molecule Ca2+ indicator by TIRFM, such as performed in the above investigation (78), may give a more precise image than measurements by epifluorescence, but it cannot reveal the fusion-relevant Ca^2+^ concentrations, postulated to exist in microdomains around Ca^2+^ channels (for an overview see (81). In these microdomains of just a few nanometers in diameter, a steep gradient exists generated by the influx of Ca^2+^ through the open channel and the buffering capacity and velocity of the cytosol. The concept resulted from research on neurotransmitter release (9, 82) and was quickly adapted to research on insulin granule exocytosis (33).

If only the high Ca^2+^ concentration within the microdomain (about 100 μM, to give a rough estimate) is sufficient to elicit the granule fusion, a clustering of pre-exocytotic vesicles or granules around the Ca^2+^ channels and the microdomains appears as a necessary consequence. This in turn entails that pre-exocytotic granules are particularly immobile and as such correspond to (a subgroup of) docked granules. In fact, clustering and immobility have been repeatedly emphasized as central features of pre-exocytotic granules (31, 33, 55). Consequently, disturbed clustering for a variety of reasons has been described as a mechanism underlying the impaired kinetics of insulin secretion in type 2 diabetes or its disease models (83–85).

The extent of glucose-stimulated insulin is not only regulated by the depolarization-induced Ca2+ influx, but also by a sequence of still incompletely understood events, which are summarized under the name “amplifying pathway” (86, for a recent review see 87). By using the genetically encoded Ca^2+^ indicator LynD3-cpv, which was targeted to plasma membrane, it could be demonstrated that no further Ca^2+^ increase occurs in the submembrane space under the conditions leading to the metabolic amplification by glucose (88). How exactly glucose stimulation amplifies the Ca^2+^-induced fusion reaction, remains an unsolved issue thus far.

To evaluate the relevance of docking, clustering and immobilization as preconditions for granule exocytosis, and to gain further insight into the mechanisms of the metabolic amplification, an experimental approach to directly measure the fusion-relevant Ca2+ concentrations in the vicinity of Ca2+ channels and insulin granules is needed. While earlier experiments brought inconclusive results in this regard (89), the current availability of GECIs with a broad spectrum of properties may enable further advances in this area (90).

## The roles of cortical actin - barrier, site of the reserve pool, site of docking or all together?

In schematic drawings cortical actin and the plasma membrane are separated by a space wider than a granule diameter (see e.g. 3, 79, 91). It is often assumed that the readily releasable pool and the reserve pool are separated by the cortical actin web which the granules of the reserve pool have to cross to replenish the readily releasable pool and to ultimately reach the fusion site. The modified model as suggested by Seino´s group (91) placed a pool of non-docked granules in the obstacle-free vicinity of the membrane-attached granule pool, theoretically enabling the restless newcomers to quickly reach the fusion sites and thus form part of the readily releasable pool. However, the relation between the non-docked granules and cortical actin remained undefined.

Support for a role of the cortical actin web not just as barrier, but as a near-membrane storage site came from experiments where instead of granuphilin another effector protein of Rab27a, exophilin8, was knocked out. Apparently, exophilin8 traps the granules into the actin network, from where they are released during stimulation (92, 93). Upon glucose stimulation, probably mediated by the Ca^2+^ rise, melanophilin dissociates granules from myosin-Va and actin in the actin cortex and by associates them with a fusion-competent, open form of syntaxin-4 on the plasma membrane. This way, a continuous supply of granules can be made available to fuse with the plasma membrane without a preceding docking period (94).

An obvious contradiction to the concept of granules being immobilized around L-type channels consists in the observation that insulin granules increase mobility, in particular lateral displacement, during the last few seconds before fusion, together with increased FRET signal of Rab 27a and its effector Slp4a (95). Similarly, pre-exocytotic granules were found to have a significantly smaller caging diameters than the non-fusing neighbours, but the caging diameter of the pre-exocytotic granules increased significantly directly before fusion (53). A plausible explanation of this late increase in mobility is that the exocytotic granule changes its position from one attachment site, possibly at the cortical actin, to another, where the actual fusion takes place.

**Figure 2:**
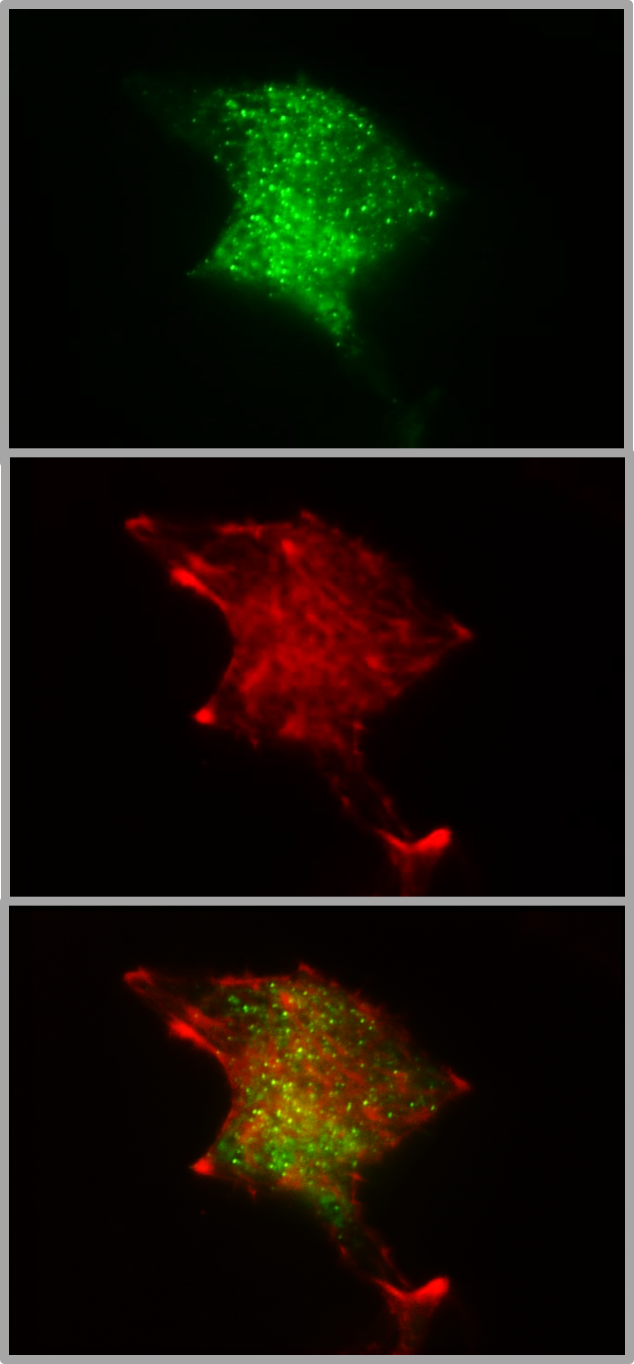
Colocalization of insulin granules and actin in the submembrane space of MIN6 cells. The insulin granules of MIN6-cells (p20 - p30) were labeled by transient transfection with hIns-EGFP and mTagRFP-T-Lifeact-7. The fluorescence emission < 560 nm (upper image) gives the conventional TIRFM image of green fluorescent granules in the submembrane space (calculated decay constant 85 nm). The fluorescence emission > 560 nm shows the presence of red-labelled actin (middle image) together with insulin granules (overlay, lower image) in the same space. The coexistence suggests that the granule mobility may reflect, at least in part, interactions with the cortical actin.

**Figure 3:**
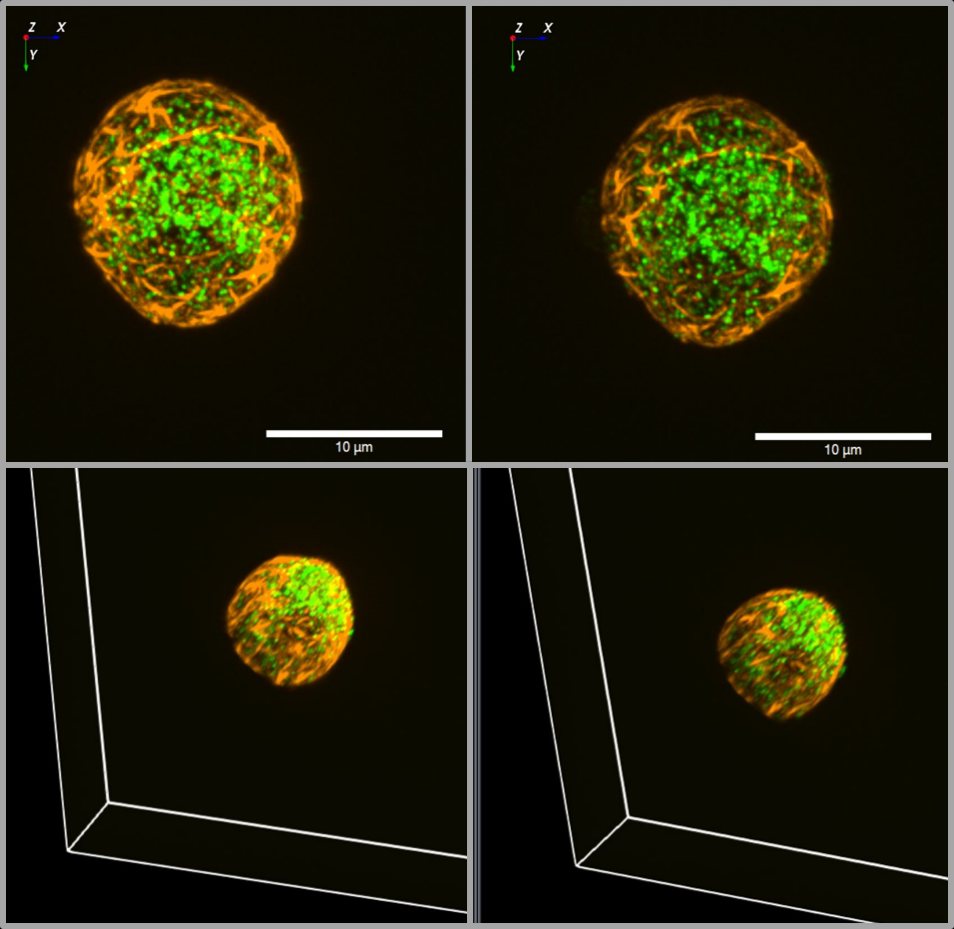
3D view of insulin granules and actin in primary beta cells. The insulin granules of primary beta cells were labeled by adenoviral transfection with hIns-EGFP (green) and mTagRFP-T-Lifeact-7 (orange). The fluorescence was excited by spinning disk CLSM (50 µm disk, 2.8fold magnification by SORA attachment, objective Nikon SR HP Apo TIRF 100x, N.A. 1.49). The z-stack was generated from 61 levels at 200 nm steps. The lower images show the same beta cell as above, but viewed from an oblique angle. The right images show the same cell as the left images after 10 min incubation in the presence of latrunculin. Note the decrease of actin by latrunculin all around the beta cell.

Most of the work on granule mobility discussed thus far has visualized the granules, but rarely together with the labeling of F-actin. Co-labelling of insulin granules with hIns-EGFP and of F-actin with mTagRFP-T-Lifeact-7, shows that both are contained within the TIRF zone when the calculated decay constant is about 80 nm, which is a typical value (**Figure 2**). The 3D image of such a dual labelled beta cell generated by spinning disk CLSM, gives even the impression that the cortical actin forms the outermost layer of the beta cell, beyond most of the granules (**Figure 3**). So, schematic drawings appear more appropriate where the cortical actin layer extends to the plasma membrane and actin cages contribute to the immobilization of granules in the immediate vicinity of the plasma membrane (see e.g. 96).

In such a model the recruiting of granules from the actin-associated reserve pool is unlikely to cause a delay in the re-filling of the readily releasable pool, which was suggested to be reason for the biphasic secretion pattern (32). Conversely, a fast reverse passage through the cortical actin web must be possible, given that 80% of the granules which are visible in the submembrane space (TIRF layer) stay there for only 1 s or less (**Table 1**). Likewise, the cumulative number of granules identified within 1 min exceeds the entire number of granules within the beta cell, a feature which can only be explained by recirculation of the granules (42).

**Figure 4:**
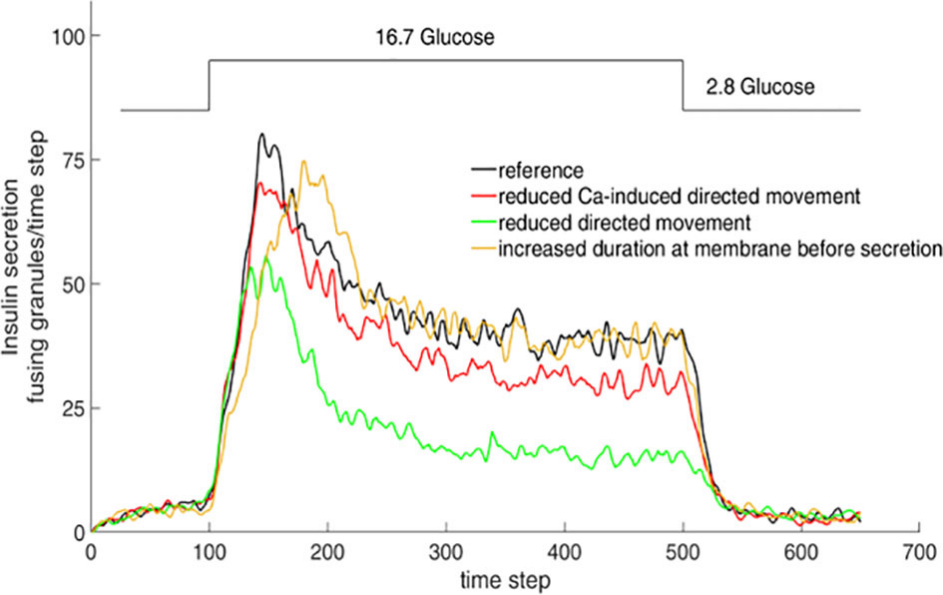
Simulation of insulin secretion as function of granule mobility. Consequences of varying the simulation parameters. The consequences were tested by halving the parameter values one by one while fixing all other parameters. When this results in the general increase of diffuse granule movement over directional movement, e.g. by lower chord lengths in the actin network, secretion is markedly reduced, even in the stationary phase (green line). Halving the parameter value describing the transition from diffuse movement into directional movement, such as generated by the increase of the cytosolic Ca^2+^ concentration, results in a similar but less pronounced decrease (red line). When the effect of halving results in the prolonged presence of the granules at the membrane prior to release, the peak value of secretion is retarded, but the stationary phase remains unchanged (yellow line). Adapted from 100.

Simulation of the interaction between insulin granules and the actin network by a cellular automaton model showed that specific features of the resulting biphasic secretion pattern could be modified by altering the cord length, the network density and the velocity of Ca^2+^ increase (**Figure 4**). While some authors have observed generalized changes in the actin pattern as a result of glucose stimulation (97, 98), others observed only spatially discrete and transient F-actin changes around each fusing granule, but no global changes (99). While the physiologically relevant changes are certainly much more discrete than the changes produced by the secretion-enhancing action of the actin-depolymerizing agent latrunculin (100, 101), there is currently no evaluation tool to quantitatively describe the structural characteristics of the cortical actin in beta cells.

An interesting new aspect of the role of cortical actin concerns the relation between endocytosis and exocytosis of insulin granules, mentioned earlier (chapter 1). Knock-out of all three dynamin genes did not only impair endocytosis but also blunted both phases of glucose-induced insulin secretion concurrent with reduced granule docking and reorganization of the cortical actin layer (102, 103). Taken together, the recent observations support a model in which submembrane cytoskeletal structures, not only the cortical actin, but also the microtubular activity (104) gate the access of the insulin granules to the plasma membrane and the exocytotic sites, thus co-regulating the secretion kinetics (105).

## Consequences of granule aging

It is generally accepted that the signals for granule generation only partially overlap with those for granule release. Granule generation is stimulated by nutrient secretagogues, but not by purely depolarizing stimuli, such as sulfonylureas or KCl-depolarization, and the glucose concentration required to stimulate insulin synthesis (and by inference, granule synthesis) is left-shifted (106). While this relation ensures that beta cell insulin stores do not become depleted, it also means that a surplus of granules is produced, which ages and has to be degraded. However, not only aged granules (those which have exceeded the half-life of 3-5 days) but also younger granules are degraded, depending on the metabolic situation of the beta cell (107, 108). So, at no time point does a signal for stimulated secretion impinge on a homogeneous granule pool.

The observation, which has mainly stimulated interest in the role of granule aging, was made decades ago by the pulse chase technique. ^3^H leucine was used to label newly synthesized insulin and it was observed by a number of groups that after a lag time, roughly corresponding to the transit time from the trans-Golgi network to the plasma membrane, the released insulin had a higher specific activity than the insulin content still present in the islets (11–13). Or, in other words, newly synthesized insulin was preferentially released.

There are two different questions associated with granule aging: First, how do the newly formed granules gain preferential access to the fusion sites? Second: is there a general mechanism of granule aging which gradually diminishes their ability to fuse and become degraded instead? An indication that the first question is related to glucose-dependent stimulus secretion coupling is the observation that the preferential release is not simply related to young age, but that the glucose concentration during the time of granule synthesis leaves a mark for preferential release (14)

Interest in this topic was revived by an observation which was made possible by the use of the timer protein (DsRedE5), which changes its fluorescence emission from green to red with a half-time of about 18 h (109). Labelling LDCVs (large dense-core vesicles) in chromaffin cells with ANF-timer fusion proteins showed that green vesicles were immobile (apparently docked) at the plasma membrane, whereas older vesicles were mobile and had a higher density in the cell interior. Moreover, nicotine stimulation released a large part of the green vesicles, but none of the red vesicles (110). Since chromaffin cells are widely considered as a model for granular exocytosis, the authors concluded that the spatial and functional segregation could be a general feature of protein-releasing LDCVs. (111).

Examining the age-dependent granule mobility in insulin-secreting cells by use of SNAP tag-labeling, led to a somewhat different result, in that young granules displayed a wide variety of mobility, whereas old (28 - 30 h) granules displayed only restricted mobility or were even immobile (112). Young immobile, but not old immobile granules could be recruited by glucose stimulation or by depolymerization of F-actin to be transported by microtubules, which may correspond to the preferential transport and release of newly synthesized insulin (112). Here, actin is considered to have mainly a retentive function for young granules and is relevant for transport parallel to the membrane, whereas axial long range transport is mediated by microtubules (49).

Another reason to consider the role of aging for granule mobility and release was the exploration of the mechanisms of desensitization by prolonged exposure to a sulfonylurea and beta cell rest by prolonged exposure to the alpha2 adrenoceptor agonist, clonidine (41). Labeling with hIns-timer showed that desensitization did not affect the proportion of aged (> 18 h), whereas rest increased it. Aged granules showed a high turnover and were under-represented in the group of long-term residents, which made up the larger part of pre-exocytotic granules. Likewise, examination of the granule content after massive stimulation of secretion showed an initial drop in the green-to-red ratio of timer-labelled granules, suggestive of a predominant release of the young, green granules (113).

**Figure 5:**
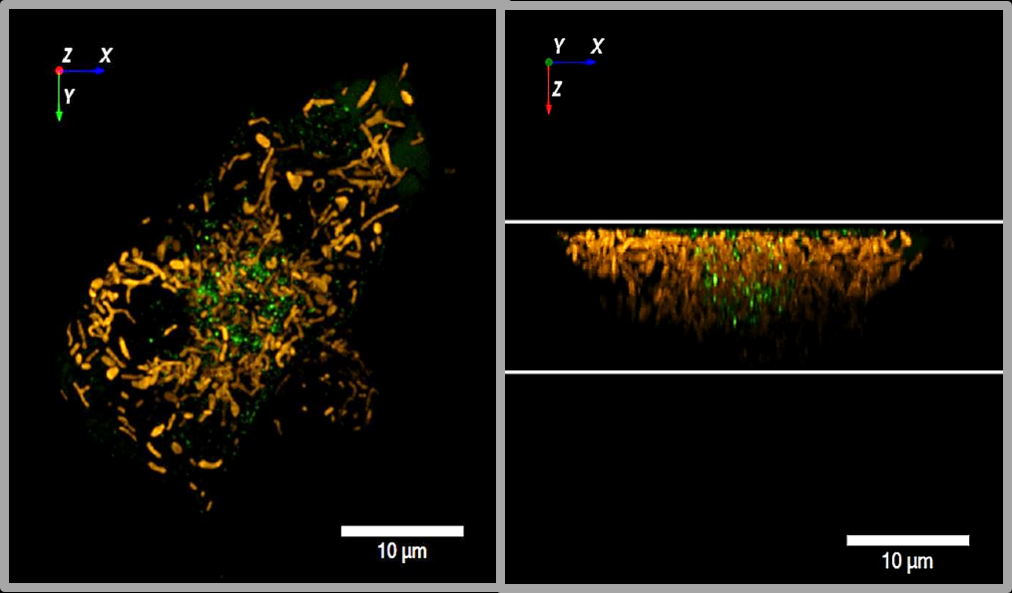
Presence of insulin granules and mitochondria in the submembrane space of MIN6 cells. The insulin granules of MIN6 cells were transiently labelled with hIns-EGFP (green) and mitochondria by loading with TMRE. The image acquisition of the z-stack was performed as in **Figure 3**. The left image shows the cell from the bottom - attachment to the cover slip, the right image shows the lateral view to demonstrate the high concentration of mitochondria in the immediate vicinity of the submembrane space.

On the balance it seems that the initial observation, obtained with neuro-endocrine chromaffin cells, that granule aging increases the mobility but at the same time decreases the ability to fuse is also valid for insulin secreting cells. It remains to be explored as to whether both features are caused by the same underlying mechanism. Further exploration of granule aging appears promising to clarify the phenomenon of metabolic memory by which the acute secretory response is influenced by preceding phases of nutrient availability or starvation (114). The energy requirement of granule mobility and local interactions with submembrane mitochondria (**Figure 5**) are factors which may be involved and need to be investigated more intensively.

## Concluding remarks

This review is focused on the increasingly complex landscape of insulin granule mobility and its contribution to the regulation of secretion. Of course, there are a number of further factors in the life time of a granule which may be of relevance for the secretory output. In many of the reports cited here a heterogeneous pattern of granule content release is observed. It has been hypothesized that a very protracted release may be the mechanism of basal insulin release (115). Likewise, the long-standing questions about the relevance of kiss-and run (or “cavicapture”) exocytoses or multigranular, compound exocytoses in beta cells (see e.g. 116, 117) are still awaiting clarification. Either mechanism would loosen the linear relation between fusion frequency and insulin output, which was indirectly supported by the demonstration of quantal ATP release during exocytosis of insulin granules (118).

Early investigations, which suggested that beta cells are polarized and insulin secretion occurs in the “advascular” part of the beta cell (119), were contradicted by 2-photon microscopy of the fluid phase tracer sulforhodamine B, which suggested that insulin granules fuse to the “abvascular” part of the beta cells in intact islets (77). Either observation raises the possibility of a localized release of insulin persisting in single isolated beta cells. Since the cell footprint makes up only about 30% of the cell surface (65) this may explain the considerable cell-to-cell heterogeneity not only with respect to the number of fusion reactions, but also with respect to the mobility pattern.

A localized release occurring not only in primary beta cells but also in MIN6 cells was rendered likely by the observation that the active zone protein, ELKS, is expressed in these cells. ELKS was organized in clusters which colocalized with syntaxin 1 clusters and (presumed) docking sites of insulin granules. The analysis of single granule mobility by TIRFM suggested that the fusions mostly occurred on the ELKS clusters. Since ELKS was found to be localized close to the plasma membrane-facing blood vessels an “advascular” release of insulin is supported (120). These data are indirectly contradicted by an investigation utilizing spinning disc CLSM to obtain a near complete 3D image of the cell within less than a second. In single MIN6 cells no clustering of fusions and no compound exocytosis could be detected by this technique (121).

Recent investigations on beta cell exocytosis within islets, however, tip the balance of evidence towards the “advascular” release hypothesis (122) and local integrin activation was suggested to target insulin secretion to the islet capillaries (123). Furthermore, it was observed that neighboring beta-cells work in synchrony and granule fusion occurred in discrete bursts during stimulation (124). The latter observation conforms with the earlier reports that the coordinated interaction of the beta-cells as occurs within the islet markedly enhances the insulinotropic efficacy of stimuli as compared with single dissociated beta-cells (125, 126). This holds also true for the comparison of single MIN6 cells vs. MIN6 cells organized in “pseudo-islets” (127, 128).

In keeping with the characteristics of TIRF microscopy, nearly all work on granule mobility was made with single cells or single cell clusters. The frequency of fusions during continuous measurements usually gave a monophasic pattern, and the assignment to first- or second phase was simply based on the temporal sequence. Given the above intra-islet interactions it may well be that a biphasic pattern in its original meaning may not be directly observable at the level of the single beta cell.

The position of beta cells within the islet may also affect granule mobility, since the focal adhesion proteins influence the organization of cortical actin (129, 130). Since the actin structure receives also input from glucose metabolism (for an overview see 131) it is a plausible hypothesis that the interaction between granules and cortical actin may result in a gating function for the admission to the sites of exocytosis. To clarify how granule age, preceding metabolic conditions (“metabolic memory”), and the signals of metabolic amplification combine to generate the appropriate secretory responses will require further advances in the quantitative 4D live cell imaging of beta cells (132).

## Author contributions

Conceptualization: RI, MM, BD. Experimentation: GB, BD, GS. Methodology: GB, BD, GS. Visualization: GB, BD, MM. Writing-original draft: RI. Writing-review and editing: GB, BD, GS, MM, RI. All authors contributed to the article and approved the submitted version.

## Funding

The authors´ research cited in this review was supported by grants from the Deutsche Forschungsgemeinschaft to RI. (Ru 368/5-2 and 5-4). Further support was obtained by grants from the Deutsche Diabetes Gesellschaft to BD and GB.

## Acknowledgments

The authors would like to thank Sabine Warmbold, Claudia Bütefisch and Verena Lier-Glaubitz for expert assistance with their work mentioned in this review.

## Conflict of interest

The authors declare that the research was conducted in the absence of any commercial or financial relationships that could be construed as a potential conflict of interest.

## Publisher’s note

All claims expressed in this article are solely those of the authors and do not necessarily represent those of their affiliated organizations, or those of the publisher, the editors and the reviewers. Any product that may be evaluated in this article, or claim that may be made by its manufacturer, is not guaranteed or endorsed by the publisher.
